# Chiisanoside Mediates the Parkin/ZNF746/PGC-1α Axis by Downregulating MiR-181a to Improve Mitochondrial Biogenesis in 6-OHDA-Caused Neurotoxicity Models In Vitro and In Vivo: Suggestions for Prevention of Parkinson’s Disease

**DOI:** 10.3390/antiox12091782

**Published:** 2023-09-20

**Authors:** Yu-Ling Hsu, Hui-Jye Chen, Jia-Xin Gao, Ming-Yang Yang, Ru-Huei Fu

**Affiliations:** 1 Graduate Institute of Biomedical Sciences, China Medical University, Taichung 40402, Taiwan; fish38@cgmh.org.tw (Y.-L.H.); huijyechen@mail.cmu.edu.tw (H.-J.C.); c1181130@gmail.com (J.-X.G.); 2 Department of Laboratory Medicine, Chang Gung Memorial Hospital, Linkou, Taoyuan 33305, Taiwan; 3 Ph.D. Program for Aging, China Medical University, Taichung 40402, Taiwan; c966111@gmail.com; 4 Translational Medicine Research Center, China Medical University Hospital, Taichung 40447, Taiwan

**Keywords:** Parkinson disease, mitochondrial biogenesis, 6-hydroxydopamine, SH-SY5Y cell, chiisanoside, PGC-1α, ZNF746, parkin, miR-181a, *C. elegans*

## Abstract

The degeneration of dopamine (DA) neurons is known to be associated with defects in mitochondrial biogenesis caused by aging, environmental factors, or mutations in genes, leading to Parkinson’s disease (PD). As PD has not yet been successfully cured, the strategy of using small molecule drugs to protect and restore mitochondrial biogenesis is a promising direction. This study evaluated the efficacy of synthetic chiisanoside (CSS) identified in the leaves of *Acanthopanax sessiliflorus* to prevent PD symptoms. The results show that in the 6-hydroxydopamine (6-OHDA) model, CSS pretreatment can effectively alleviate the *reactive oxygen species* generation and apoptosis of SH-SY5Y cells, thereby lessening the defects in the *C. elegans* model including DA neuron degeneration, dopamine-mediated food sensitivity behavioral disorders, and shortened lifespan. Mechanistically, we found that CSS could restore the expression of proliferator-activated receptor gamma coactivator-1-alpha (PGC-1α), a key molecule in mitochondrial biogenesis, and its downstream related genes inhibited by 6-OHDA. We further confirmed that this is due to the enhanced activity of parkin leading to the ubiquitination and degradation of PGC-1α inhibitor protein Zinc finger protein 746 (ZNF746). Parkin siRNA treatment abolished this effect of CSS. Furthermore, we found that CSS inhibited 6-OHDA-induced expression of miR-181a, which targets parkin. The CSS’s ability to reverse the 6-OHDA-induced reduction in mitochondrial biogenesis and activation of apoptosis was abolished after the transfection of anti-miR-181a and miR-181a mimics. Therefore, the neuroprotective effect of CSS mainly promotes mitochondrial biogenesis by regulating the miR-181a/Parkin/ZNF746/PGC-1α axis. CSS potentially has the opportunity to be developed into PD prevention agents.

## 1. Introduction

Parkinson’s disease (PD), the second most commonly diagnosed neurodegenerative disorder in hospitals, usually occurs in older adults. The main symptoms are motor deficits and, later cognitive and behavioral impairments. Mitochondrial dysfunction has been shown in numerous studies to contribute to the cytopathic features of PD [1]. These include decreased activity of mitochondrial complex I, loss of mitochondrial DNA, increased mitochondrial ROS, and a number of mitochondrial damage observed in dopamine neurons (DA) in the substantia nigra of PD patients [2]. The balance of mitochondrial biogenesis and mitophagy maintains mitochondrial homeostasis [3]. Mitochondrial biogenesis is tightly coupled to several cellular programs, such as cell growth, differentiation, and death. Therefore, maintaining the fitness of mitochondrial biogenesis in DA neurons is important to prevent the establishment of PD [4].

Peroxisome proliferator-activated receptor gamma coactivator 1 α (PGC-1α, Gene ID: 10891) is a strong transcriptional coactivator that mainly mediates the expression of genes related to mitochondrial biogenesis and energy metabolism induced by external physiological stimuli [5]. PGC-1α synergistically regulates the activity of cAMP response element-binding protein (CREB) and nuclear respiratory factors (NRFs) by binding to nuclear receptor PPAR-γ and affects the expression of downstream genes, including mitochondrial transcription factor A (TFAM, Gene ID: 7019) and nuclear respiratory factor 1 (NRF1, Gene ID: 4899) [6]. PGC-1α has been considered a potential therapeutic target in PD as it maintains normal mitochondrial metabolism [7]. TFAM is a key transcription factor for mitochondrial genes and is also involved in the packaging, stability, and replication of the mitochondrial genome [8]. A homodimeric NRF1 transcription factor regulates the expression of metabolic genes involved in cellular respiration and nuclear genes required for mitochondrial DNA replication and transcription and is also involved in the growth of synapses [9]. Thus, the activity of TFAM and NRF1 has a critical impact on the survival of DA neurons under stress.

The transcriptional expression of PGC-1α is tightly regulated by many factors, among which zinc finger protein 746 (ZNF 746, Gene ID: 155061) acts as a negative regulator of activity [10]. ZNF 746 mediated inhibition of transcriptional expression of PGC-1α causes disruption of mitochondrial homeostasis and dysfunction, ultimately leading to DA neuronal death [11]. To maintain neuronal survival under normal conditions, parkin (PARK2, Gene ID: 5071), an E3 ubiquitin ligase, can ubiquitinate intracellular ZNF746 protein and promote ZNF746 degradation via the ubiquitin-proteasome system (UPS) [12]. However, when parkin activity is deficient, ZNF746 will repress the transcription of PGC-1α by binding to the insulin response sequences in the promoter region, resulting in impaired mitochondrial biogenesis, further causing the death of DA neurons. [13]. Parkin also promotes the activation of autophagy and proteasomes to clear damaged mitochondria [14,15]. Other studies have shown that parkin prevents apoptosis to increase the survival of damaged or aged cells [16]. In addition, some patients of PD are associated with parkin mutations [17]. Furthermore, those mutations enhance ZNF746 protein expression in neurons of patients with PD [13].

MicroRNA (miRNA) is a post-transcriptional regulator that matures into a short single-stranded RNA (~22 nt), and its dysregulation in the pathogenesis of various disorders has been widely reported [18]. miRNA can complement the coding (CDSs) and non-coding (UTR) sequences on the mRNA of the target gene and inhibit the expression of the target gene via translational repression or degradation of mRNA [19]. PD pathogenesis has been connected with some specific miRNAs that are involved in the molecular mechanisms of mitochondrial function, apoptosis, neuroinflammation, pyroptosis, neuronal survival, and autophagy [20].

**Figure 1:**
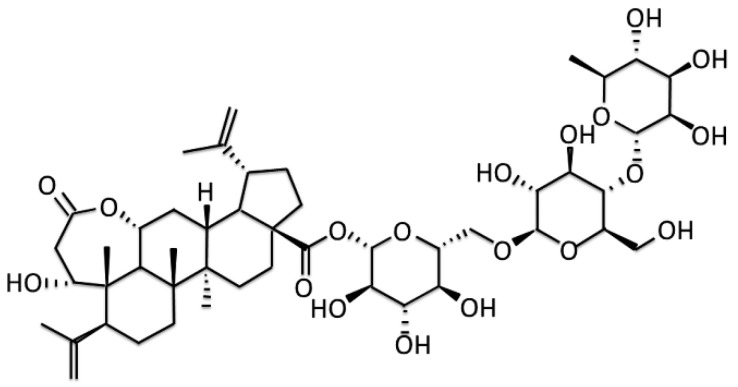
Molecular structure diagram of chiisanoside (CSS) from leaves of *Acanthopanax sessiliflorus*.

So far, there is no effective method to delay the process and cure PD. It is an important research direction in this field to screen and characterize small molecular compounds for delaying or treating PD. Chiisanoside (CSS, Figure 1) is a lupine triterpenoid extracted from the leaves of Acan-thopanax sessiliflorus and has various important pharmacological activities, including anti-oxidation [21], anti-inflammatory [21,22], anti-depression [23], anti-obesity [24], and anti-platelet aggregation [25]. In the aconitine-induced cardiomyocyte injury model, CSS can lessen cell apoptosis by decreasing ROS production, maintaining cellular calcium ion balance, downregulating the expression of cleaved caspase-3 and Bax, and enhancing the expression of Bcl-2 [22]. In addition, CSS can increase the activities of superoxide dismutase, catalase, and glutathione in treating LPS/D-GalN-induced acute liver injury. On the contrary, CSS inhibits malondialdehyde activity and the expression of inflammation-related factors. These properties may be achieved by promoting the nuclear translocation of nuclear factor erythroid 2-related factor 2 and the expression of oxygenase-1 and inhibiting NF-κB phosphorylation [21]. In a mouse model of depression, CSS treatment augmented DA and gamma-aminobutyric acid (GABA) levels in the mouse brain. Similarly, the expression of brain-derived neurotrophic factor and tropomyosin-related kinase B in the hippocampus was upregulated [23]. Currently, the most commonly used pharmacological model of PD is treatment with the neurotoxin 6-hydroxydopamine (6-OHDA). It specifically arrives at DA neurons via the transport of dopamine active transporter (DAT) to carry out autoxidation or cause damage to complexes I and IV of the mitochondrial electron transport chain, resulting in the formation of oxidative stress of ROS (mainly superoxide and hydrogen peroxide) [26]. Intracellular ROS can damage and promote apoptosis of DA neurons. Finally, it causes behavioral deficits associated with chronic dopamine insufficiency in animal models [27]. In addition, SH-SY5Y cells [28] and *Caenorhabditis elegans* (*C. elegans*) [29,30] are simple in vitro and in vivo model systems for preliminary PD mechanism research and drug screening platforms. Because CSS has properties of antioxidant, anti-inflammation, and increasing dopamine in the brain, these properties are associated with efficacy against the cytopathological features of PD. However, whether CSS has a therapeutic benefit in PD via these properties has not yet been proven. Here, we used SH-SY5Y cells and *C. elegans* animal models to explore the neuroprotective feature of CSS against 6-OHDA neurotoxicity and α-synuclein accumulation. Furthermore, we deeply studied the possible mechanism of action of CSS from the perspective of regulating mitochondrial biogenesis.

## 2. Materials and Methods

### 2.1. Chemicals and Maintenance/Treatment of SH-SY5Y Cell Line

Synthetic Chiisanoside (CSS, mol. wt. 955.1, 98% purity) was obtained from Rainbow Biotechnology Co (Taipei, Taiwan). Other chemicals were purchased from Sigma-Aldrich (St. Louis, MO, USA) unless otherwise noted. The SH-SY5Y cell line was a gift from Dr. Chia-Wen Tsai (Chinese Medical University, Taichung, Taiwan). Fetal bovine serum (FBS), culture medium, and supplements were purchased from Gibco, Thermo Fisher Scientific (Waltham, MA, USA). Cell culture, subculture, and storage refer to the cell culture guide of ATCC (CRL-2266). Except for specific experiments, SH-SY5Y cells (2.5 × 10^6^) were generally inoculated in 60 mm culture dishes and medium containing 1 or 2 μM CSS for 24 h. Then, they were exposed to 100 μM 6-OHDA for 12 h (Western blotting) or 18 h (general assay).

### 2.2. Determination of SH-SY5Y Cell Viability

SH-SY5Y cells (4 × 10^3^) were inoculated in 96-well culture plates and then treated with as described in Section 2.1. Next, CellTiter-Blue^®^ Reagent (Promega, Madison, WI, USA) was mixed with the fresh medium to determine the viability of the cells according to the instruction manual.

### 2.3. Measurement of Mitochondrial Membrane Potential in SH-SY5Y Cells

SH-SY5Y cells were treated as described in Section 2.1. Then, cells were washed with PBS, followed by the addition of fresh medium containing 1 μM DiOC6 dye. After 30 min of reaction, the change in mitochondrial membrane potential (green fluorescence) was detected and quantified.

### 2.4. TUNEL Assay of SH-SY5Y Cells

SH-SY5Y cells were treated as described in Section 2.1. Then, we used the Click-iT™ Plus TUNEL assay kit (Invitrogen, Carlsbad, CA, USA) to detect apoptosis of cells in situ according to the instruction manual.

### 2.5. Flow Cytometry of FITC-Tagged Annexin-V and Propidium Iodide Staining on Apoptosis of SH-SY5Y Cells

SH-SY5Y cells were treated as described in Section 2.1. Apoptosis detection was performed using the FITC Annexin-V Apoptosis Detection Kit I (BD Biosciences Pharmingen, San Diego, CA, USA) according to the instruction manual. The collection gate per sample was 10,000 events.

### 2.6. Protein Expression Detection by Western Blotting in SH-SY5Y Cells

SH-SY5Y cells were treated as described in Section 2.1. The cell extract was isolated by electrophoresis of SDS-polyacrylamide gels. Then, the gel is transferred to PVDF membranes. After that, specific antibodies were added and reacted with membranes. The following day, the membranes were washed, and HRP-conjugated secondary antibodies were added. After 1 h at room temperature, the enhanced chemical light emission method was used to detect the location and intensity of the specified proteins in membranes. ZNF746 antibody was purchased from Sigma-Aldrich. Other antibodies were purchased from Cell Signaling Technology (Beverly, MA, USA) unless otherwise stated.

### 2.7. Quantification of Intracellular Reactive Oxygen Species in SH-SY5Y Cells

SH-SY5Y cells were treated as described in Section 2.1. Cells (5 × 10^3^) were incubated in black 96-well plates with H2DCFDA (25 μM) for 30 min in the dark. Then, the cells were washed, and the fluorescence signal in samples was detected using a microplate reader.

### 2.8. *Mitochondrial Staining in SH-SY5Y Cells*

MitoTracker™ Green FM purchased from Cell Signaling Technology was dissolved in DMSO to prepare a 1 mM stock solution. SH-SY5Y cells were treated as described in Section 2.1. MitoTracker™ Green FM was mixed with the fresh medium at a concentration of 500 nM. After 20 min of reaction at 37 °C, the samples were analyzed by flow cytometry analysis (Excitation: 490 nm, Emission: 516 nm) or fluorescence microscopy to observe the content of cellular *mitochondria*.

### 2.9. Citrate Synthase *Activity Assay of Mitochondrial in SH-SY5Y Cells*

We performed this analysis according to the manufacturer’s instructions for the Citrate Synthase Assay Kit (Sigma-Aldrich). First, the cells were washed and lysed using low-strength complete formula RIPA buffer (Thermo Fisher Scientific) to obtain whole cell extracts. Next, we added 50 μg of sample to the reaction mixture (including 1× assay buffer, 30 mM acetyl CoA solution, 10 mM DTNB solution, and 10 mM OAA solution) and mixed evenly. After 1.5 min, the mixture was measured using a spectrophotometer (412 nm).

### 2.10. Immunoprecipitation (IP) Assay for SH-SY5Y Cells

SH-SY5Y cells were treated as described in Section 2.1. Cells were lysed in EBC buffer and centrifuged, and then the soluble supernatant was collected. Next, we cleared the supernatant with protein A-sepharose and added anti-ZNF746 antibody or normal rabbit immunoglobulin G (Cell Signaling Technology) to the supernatant to incubate overnight at 4 °C. The following day, the supernatant was added with A-sepharose beads (0.1 g/L) and reacted for 4 h at 4 °C. Finally, the beads were washed with EBC buffer and the phosphate buffer. Immunoprecipitation complexes were obtained by centrifugation (14,600× *g*) for 10 min at 4 °C and then assayed for ubiquitin level by Western blotting.

### 2.11. Transient Transfection of Small Interfering RNAs in SH-SY5Y Cells

We used Lipofectamine 2000 transfection reagent (Invitrogen) to transfect siRNA (75 nM) of parkin or nontargeted control according to the product instructions Cells were inoculated on 6-well culture plates (2.0 × 10^5^ cells). When 70% confluence is reached, transfection is performed for 24 h. Cells were then treated as described in Section 2.1. The siRNA sequence targeting human parkin is 5′-UUCGCAGGUGACUUUUCCU CUGGUCA-3′. The control siRNA sequence is 5′-AAUUCUCCGAACGUGUCACGU-3′.

### 2.12. Reverse Transcriptase–Real-Time Polymerase Chain Reaction (RT-qPCR) for Parkin Gene in SH-SY5Y Cells

SH-SY5Y cells were treated as described in Section 2.1. Total RNA was obtained from the cells using TRIzol reagent (Invitrogen), and the reaction mixture with RNA was incubated at 55 °C for 60 min for cDNA synthesis. For identification of the mRNA expression of parkin, PCR was implemented. An initial cycle of 94 °C, 10 min was performed, and then PCR reactions: 35 cycles of 94 °C for 15 s and 58 °C for 1 min. Fold changes in mRNA levels were quantified using the comparative 2^−ΔΔCT^ method with GAPDH mRNA as an endogenous control. The primer sequences for parkin were 5′-CCAGAGGAAAGTCACCTGCGAA-3′ (forward) and 5′-CTGAGGCTTCAAATACGGCACTG-3′ (reverse). GAPDH were 5′-AGCCACATCGCTCAGACAC-3′ (forward) and 5′-GCCCAATACGACCAAATCC-3′ (reverse).

### 2.13. Measuring the Expression Level of MiR-181a by RT-qPCR in SH-SY5Y Cells

SH-SY5Y cells were treated as described in Section 2.1. We used a High Pure miRNA Isolation Kit (Roche diagnostics, GmbH, Mannheim, Germany) to isolate miRNA according to the product description. The concentration of the extracted RNA was calculated using an ultraviolet spectrophotometer. miRNA was reverse transcribed using the MystiCq microRNA cDNA Synthesis Mix kit (Sigma-Aldrich). Finally, we used the MystiCq microRNA qPCR Assay System (Sigma-Aldrich) and the ABI StepOnePlus system (Applied Biosystems, Inc) to detect miR-181a expression (mature sequence: AACAUUCAACGCUGUCGGUGAGU) in the cell lines. An initial cycle of 94 °C, 10 min was performed, and then PCR reactions: 35 cycles of 94 °C for 20 s, 68 °C for 30 s, and 72 °C for 30 s. U6 was taken as a reference gene. The expression quantity was calculated by 2^−ΔΔCq^ method.

### 2.14. Treatment of Human MiR-181a Inhibitors and Mimics in SH-SY5Y Cells

Has-mir-181a inhibitors, its negative controls, and has-mir-181a mimics were designed with reference to a previous report [31]. iR-181a inhibitor sequence: 5′-ACUCACCGACAGCGUUGAAUGUU-3′. Negative controls: 5′-UUCUCCGAACGUGUCACGUTT-3′. miR-181a mimic sequence:5′-AACAUUCAACGCUGUCGGUGAGU-3′. According to the product attachment, we used Lipofectamine 2000 (Invitrogen) to transiently transfect the inhibitor, its negative control, or mimics into cells. The concentration was 50 µM, and the transfection efficiency was confirmed using All-Stars cell death control (Qiagen, Germantown, MD, USA). After 12 h of transfection, cells were treated as described in Section 2.1.

### 2.15. Maintenance and Synchronization of Nematodes (C. elegans)

The Caenorhabditis Genetics Center (University of Minnesota, St. Paul, MN, USA) academically provides us with the following experimental resources: (1) wild-type Bristol N2 strain; (2) transgenic BZ555 strain (Pdat-1::GFP); (3) transgenic N5901 strain (Punc-54::α-Syn::YFP); (4) transgenic DA2123 strain (Plgg-1::GFP::lgg-1); (5)VC1024 strain [pdr-1 (gk448)]; and (6) *E. coli* strain OP50. We used the previously described protocol for general culture and synchronization of nematodes [32].

### 2.16. Food Clearance Test to Choice the Concentration of CSS for Nematode Treatment

In the food clearance test, we diluted CSS continuously to the indicated concentration in S medium and then added the overnight incubated OP50 to the medium. Following, CSS/OP50/S medium and approximately twenty L1 stage nematodes were loaded into a 96-well plate. Lastly, we measured the value of OD_600_ of the cultures daily for 6 days.

### 2.17. CSS Pretreatment and 6-OHDA Exposure in Nematodes

The L1 stage nematodes were shifted to CSS/OP50/NGM medium for 24 h at 22 °C to reach the L3 stage and then reacted with 6-OHDA (50 mM)/ascorbic acid (10 mM) solution for 1 h. Lastly, the nematodes were washed and moved to CSS/OP50/NGM/FUDR plates for incubation. After 3 days, the nematodes were ready for analysis for various experiments.

### 2.18. The Analysis of DA Neuron Degeneration in Nematodes

The BZ555 nematodes were treated as described in Section 2.17. Fluorescence imaging of head DA neurons was detected, and fluorescence intensity was analyzed. In addition, we used the observation to assess whether DA neurons degenerated in nematodes. If dendrites bubbled or disappeared, it was determined that the DA neurons were degenerating.

### 2.19. Food-Sensitive Behavior Test in Nematodes

We first prepared a 9 cm NGM assay plate. *E. coli* OP50 was spread within a radius between 4.5 cm and 0.5 cm. The N2 nematodes were treated as described in Section 2.17. We washed and dropped the well-fed nematodes onto the center of the assay plate. After 5 min, the movement rate (S-type frequency) of the nematodes on the empty lawn and the bacterial lawn was tested three times at intervals of 20 s. The slowing rate = (Movement rate in the empty lawn-movement rate in the bacterial lawn)/Movement rate in the empty lawn. The average slowing rate of 20 nematodes per group was calculated.

### 2.20. Lifespan Evaluation in Nematodes

The L1 stage nematodes were shifted to CSS/OP50/NGM medium for 24 h to reach L3 stage and exposed to 6-OHDA solution for 1 h. Then, the nematodes were moved to CSS/OP50/NGM/FUDR plates for incubation. The nematodes were changed to fresh plates every 3 days until all nematodes died. The number of surviving nematodes was calculated daily. Dead nematodes were considered as those that did not sense repeated contact with the worm picker. However, nematodes that migrated to the wall or disappeared due to dehydration were omitted from the analysis. Survival curves were acquired using the Kaplan–Meier method and SPSS software Version 12 (IBM, Armonk, NY, USA).

### 2.21. Determination of Reactive Oxygen Species Level in Nematodes

The N2 nematodes were treated as described in Section 2.17. Following the steps in Section 2.7, we collected and washed thirty nematodes of each group and shifted them to a 96-well plate containing PBS. Next, H2DCFDA was added, and the fluorescence values were measured every 15 min for 150 min reaction time using a microplate reader.

### 2.22. Isolation of Total RNA and Determining of Gene Expression in Nematodes

The N2 nematodes were treated as described in Section 2.17. We isolated total RNA from nematodes using TRIzol reagents (Invitrogen) and glass beads according to the earlier described experimental process [33]. RT-qPCR analysis was used to quantify gene expression as described in Section 2.11. The primer sequences for *Pdr-1* were 5′-TGCTCGTCAACCTCTGTTC-3′ (forward) and 5′-TCACTTTCTCCTTCCCATCAC-3′ (reverse); F45E4.9 were 5′-CGTGCTTCTGTCGCAGCTTC-3′ (forward) and 5′-CCCATTTCTGGAGGACGACA-3′ (reverse). SKN-1a were 5′-GTTCCCAACATCCAACTACG-3′ (forward) and 5′-TGGAGTCTGACCAGTGGATT-3′ (reverse). Analyses were implemented using the comparative 2^−ΔΔCt^ method, and ploidy differences were calculated using act-1 expression as an endogenous control.

### 2.23. Analysis of a-Synuclein Accumulation in Nematodes

The L1 stage of NL5901 nematodes was treated CSS for 3 days and then fixed on a slide (containing 2% agar pad and sodium azide) following covered with a coverslip. The imaging of body muscle cells of the nematode was obtained by fluorescence microscope, and signal intensity was assessed using ImageJ software (version 1.53, National Institutes of Health, Bethesda, MD, USA).

### 2.24. Detection of Autophagy Activity in Nematodes

DA2123 nematodes were used to observe and evaluate autophagy activity. L1 stage DA2123 nematodes were treated CSS for 3 days, and then the positive puncta (green dots) in the seam cells of the outer epidermal was counted.

### 2.25. Statistical Methods in Experiments

Statistical Methods in experiments were performed using commercially available SAS software Version 9.4 (SAS Institute Inc., Cary, NC, USA). Each testing was conducted three times. Data are revealed as mean ± standard deviation (SD). Statistical significance was acquired using one-way ANOVA and Tukey’s post hoc test. A *p* value < 0.05 was considered a significant difference.

## 3. Results

### 3.1. Chiisanoside (CSS) Inhibited Apoptosis in the SH-SY5Y Cell Model Exposed to 6-OHDA

**Figure 2:**
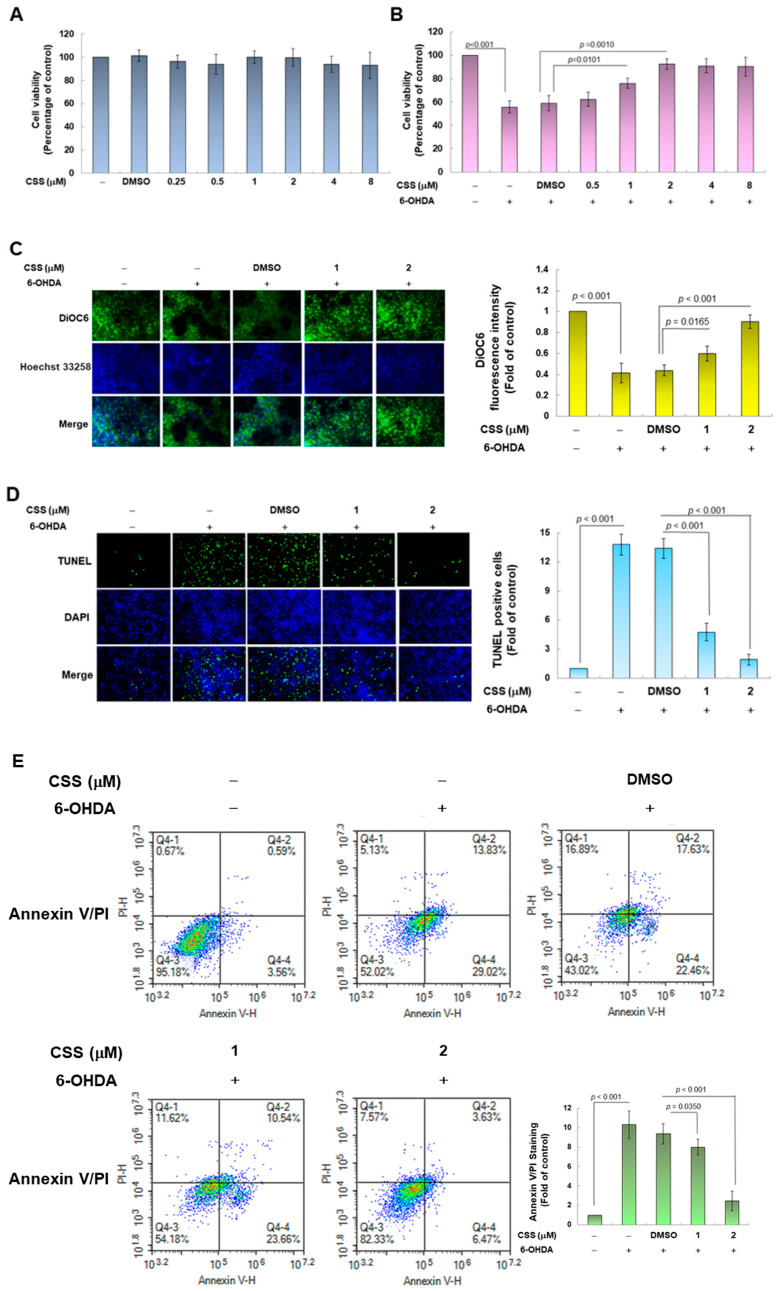

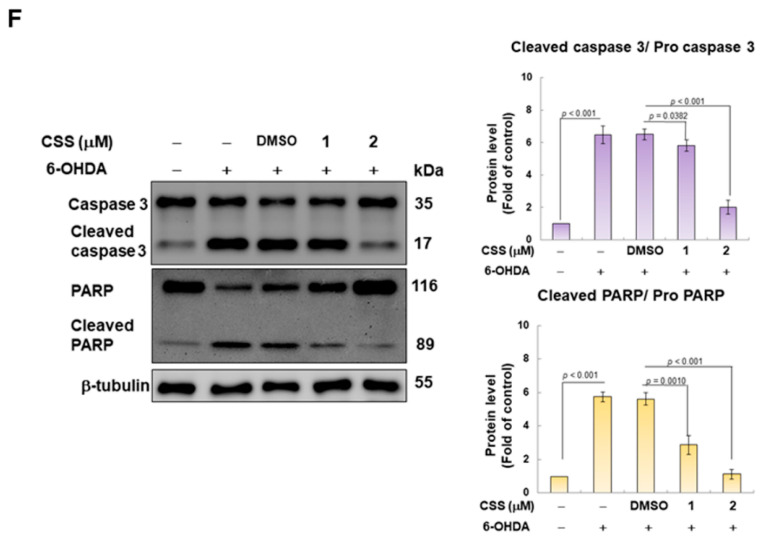
6-OHDA-induced apoptosis of SH-SY5Y cells could be alleviated by CSS pretreatment. (**A**) SH-SY5Y cells pretreated with different concentrations of CSS. After 24 h, the cell viability was detected by CellTiter Blue Cell Viability Assay. (**B**) Cells from (**A**) were further exposed to 6-OHDA (100 μM) for 18 h before cell viability analysis. (**C**–**F**) SH-SY5Y cells were pretreated with CSS for 24 h and exposed to 6-OHDA for 18 h (**C**–**E**) or 12 h (**F**). (**C**) Changes in mitochondrial membrane potential were detected by DiOC6 staining via fluorescence microscopy (200×). (**D**) The proportion of cells with DNA fragmentation was assessed by fluorescence microscopy (100×) using the TUNEL assay. (**E**) The number of apoptotic cell populations was quantified by flow cytometry with annexin V-FITC conjugated and propidium iodide (PI) staining. (**F**) The levels of apoptosis-related proteins were measured by Western blot. The histogram is the result of a quantitative analysis of the measured signal using ImageJ software (version 1.53). Use β-tubulin to unify the loading level of total protein in each group.

To assess the neuroprotective effect of CSS, we used the SH-SY5Y cell model exposed to neurotoxin 6-OHDA. First, we employed the CellTiter Blue Cell Viability Assay to confirm the appropriate concentration of CSS treatment. Cells were treated with a series of different concentrations of CSS for 24 h before analysis. The results showed that CSS concentrations lower than 8 μM had no significant effect on cell viability (Figure 2A). If treated cells were further exposed to 6-OHDA (100 μM) for 18 h. The results showed that 6-OHDA-induced cell death was dose-dependently decreased in 0.5 to 2 μM CSS pretreatment (Figure 2B). Pretreatment with 2 μM CSS increased cell viability 1.6-fold (*p* = 0.001) compared to the DMSO/6-OHDA group. In addition, CSS concentrations higher than 2 μM did not enhance the neuroprotective effect of CSS. Therefore, we used 1 and 2 μM CSS on cell models in subsequent experiments.

Next, we evaluated the protective effect of CSS on 6-OHDA-induced apoptosis. First, we observed the change in mitochondrial membrane potential (MMP) by DiOC6 staining. 6-OHDA exposure impaired MMP by 59% compared with the control group (*p* < 0.001) (Figure 2C). However, compared with the DMSO/6-OHDA group, 2 μM CSS pretreatment could increase MMP by 2.0-fold (*p* < 0.001, Figure 2C). Second, we used the TUNEL assay to reflect the situation of apoptosis by analyzing the fragmentation of chromatin DNA. The results showed that compared with the control group, 6-OHDA exposure increased cellular DNA fragmentation by 13.8-fold (*p* < 0.001) (Figure 2D). In the 2 μM CSS pretreatment group compared with the DMSO/6-OHDA group, the cellular DNA fragmentation decreased by 86.2% (*p* < 0.001) (Figure 2D). Third, we used double staining of Annexin V-FITC and PI to analyze the number of apoptotic cell populations by flow cytometry. The results showed a 10.3-fold increase in the apoptotic cell population in the 6-OHDA-exposed group compared with the control group (*p* < 0.001) (Figure 2E). Whereas after 2 μM CSS pretreatment, the apoptotic cell population was reduced by 73.4% compared with the DMSO/6-OHDA group (*p* < 0.001) (Figure 2E).

Finally, we performed activity analysis of apoptosis-related proteins by Western blot. The results showed that under 6-OHDA exposure, the ratios of cleaved caspase 3/procaspase 3 and cleaved PARP/pro-PARP increased by 6.5-fold (*p* < 0.001) and 5.7-fold (*p* < 0.001), respectively (Figure 2F). Under 2 μM CSS pretreatment, compared with the DMSO/6-OHDA group, the ratios of cleaved caspase 3/pro caspase 3 and cleaved PARP/pro-PARP decreased by 69.2% (*p* < 0.001) and 80.4% (*p* < 0.001) (Figure 2F). According to the above analysis, CSS pretreatment at 1 and 2 μM has a significant dose-dependent improvement effect on SH-SY5Y cell apoptosis induced by 6-OHDA.

### 3.2. CSS Pretreatment Reduces the Generation of Cellular ROS Induced by 6-OHDA and Increases the Biogenesis of Mitochondria in the SH-SY5Y Cell Model

**Figure 3:**
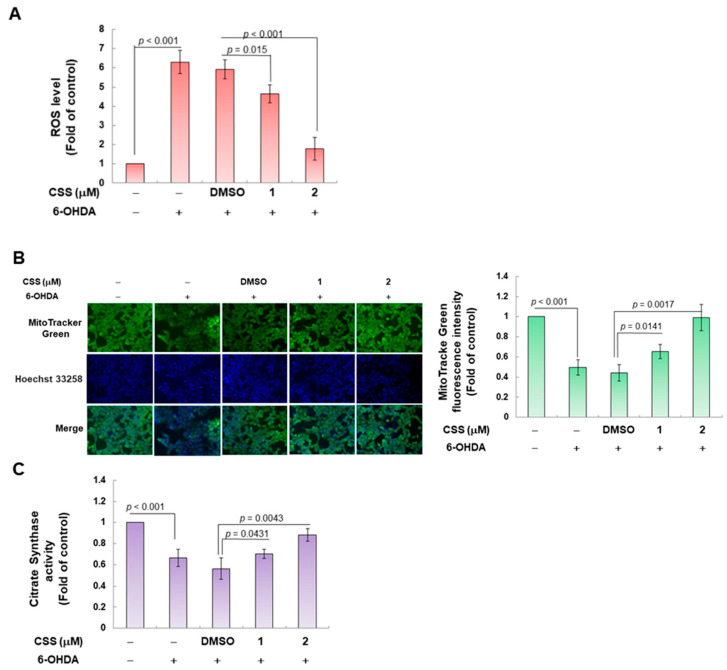

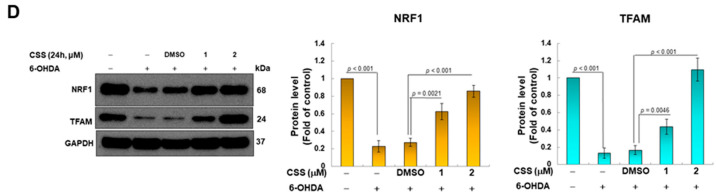
CSS pretreatment reduced the generation of cellular ROS and increased the biogenesis of mitochondria in the 6-OHDA-exposed SH-SY5Y cell model. After 24 h of CSS pretreatment, cells were exposed to 100 μM 6-OHDA for an additional 18 h. (**A**) The ROS content in cells was measured using an H2DCFDA probe by a microplate reader. (**B**) The mitochondrial content in the cells was stained with MitoTracker and observed with a fluorescent microscope. Fluorescence intensity was quantified by ImageJ software. (**C**) The mitochondrial content in the cells was quantified by citrate synthase activity assay. (**D**) After CSS pretreatment for 24 h, SH-SY5Y cells were exposed to 100 μM 6-OHDA for 12 h, and finally, the expression of mitochondrial biogenesis-related genes NRF1 and TFAM was evaluated by Western blotting. The histogram is the result of a quantitative analysis of the measured signal using ImageJ software (version 1.53). The internal loading control was using GAPDH.

6-OHDA induces ROS production in DA neurons and SH-SY5Y cells [34]. Our analysis showed that CSS dose-dependently reduced ROS levels in cells exposed to 6-OHDA (Figure 3A). Compared with the DMSO/6-OHDA group, pretreatment with 2 μM CSS reduced the level of ROS by about 69.5% (*p* < 0.001) (Figure 3A). Since earlier studies have shown that 6-OHDA-induced ROS production is associated with mitochondrial damage and loss [35], we further analyzed the changes in mitochondrial content by MitoTracker staining in SH-SY5Y cells after 6-OHDA exposure. The results displayed that the mitochondrial content was significantly reduced by 50.3% after 6-OHDA exposure compared with the control group (*p* < 0.001) (Figure 3B). Conversely, after pretreatment with 2 μM CSS, compared with the DMSO/6-OHDA group, the mitochondrial content increased 2.2-fold (*p* = 0.0017) (Figure 3B). In addition, we analyzed citrate synthase activity, which more accurately reflects mitochondrial content. The results in Figure 3C showed that compared with the control group, the enzyme activity in the 6-OHDA group decreased by 33.7% (*p* < 0.001). However, CSS can concentration-dependently improve the activity of citrate synthase down-regulated by 6-OHDA. Compared with the DMSO/6-OHDA group, citrate synthase activity increased 1.6-fold (*p* = 0.0043) under 2 μM CSS treatment (Figure 3C). Thus, CSS can significantly recover the mitochondrial content of SH-SY5Y cells damaged by 6-OHDA.

### 3.3. CSS Pretreatment Can Increase the Expression of Genes Related to Mitochondrial Biogenesis in SH-SY5Y Cells

Since our findings showed that 6-OHDA exposure decreased intracellular mitochondrial content, conversely, CSS pretreatment increased intracellular mitochondrial content. Therefore, we wanted to further confirm the role of CSS in enhancing mitochondrial biogenesis in 6-OHDA-exposed cells. It is known that the expression of NRF1 and TFAM is closely connected to the activity of mitochondrial biogenesis [36]. We next analyzed the intracellular expression of both. According to Western blot analysis, the protein expressions of NRF1 and TFAM were lessened by 77.3% (*p* < 0.001) and 86.7% (*p* < 0.001) in 6-OHDA-exposed cells, respectively, compared with the control group (Figure 3D). However, the inhibitory effect of 6-OHDA on the expression of NRF1 and TFAM proteins was reversed under CSS pretreatment (Figure 3D). The protein expression of NRF1 and TFAM was augmented by 3.1-fold (*p* < 0.001) and 6.6-fold (*p* < 0.001) under 2 μM CSS group compared with the DMSO/6-OHDA group, respectively (Figure 3D).

### 3.4. 6-OHDA Induces the Inhibition of ZNF746 on PGC-1α, Which Can Be Prevented by CSS Pretreatment

**Figure 4:**
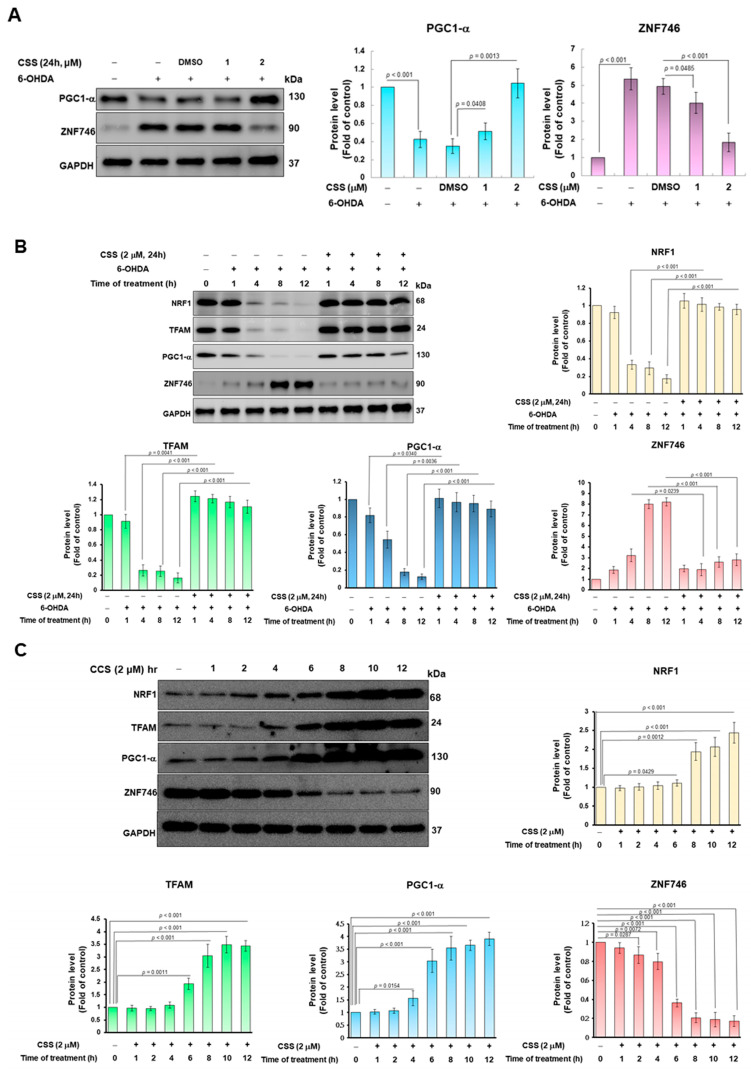
CSS pretreatment diminished the upregulation of ZNF746 expression and the downregulation of PGC-1α expression in SH-SY5Y cells caused by 6-OHDA exposure. (**A**) Protein expression of PGC-1α, ZNF746, NRF1, and TFAM was quantified by Western blotting in cells pretreated with CSS (24 h) and then exposed to 6-OHDA (100 μM, 12 h). The protein normalized for sample loading was GAPDH. (**B**) The 6-OHDA exposure times in (**A**) were subdivided into 0, 1, 4, 8 and 12 h. (**C**) The protein expression of PGC-1α, ZNF746, NRF1, and TFAM was analyzed and quantified by Western blot after cells were treated with CSS at the indicated time. The protein loading control was GAPDH.

Previous studies showed that the gene expression related to mitochondrial biogenesis was positively correlated with PGC-1α transcription factor activity. Conversely, ZNF746 prevents mitochondrial biogenesis by repressing PGC-1α expression [37]. Therefore, we wanted to observe the effect of CSS pretreatment on the expression of PGC-1α and ZNF746 in SH-SY5Y cells exposed to 6-OHDA. First, we analyzed the protein expression of PGC-1α and ZNF746 in SH-SY5Y cells exposed to 6-OHDA (100 μM) for 12 h. The results showed that 6-OHDA exposure reduced the expression of PGC-1α in the cells by 57.7% compared with the control group (*p* < 0.001). However, 6-OHDA increased the expression of ZNF746 by 5.4-fold (*p* < 0.001, Figure 4A). However, under 2 μM CSS pretreatment, compared with the DMSO/6-OHDA group, the expression of PGC-1α in the cells increased 3.0-fold (*p* < 0.001). In contrast, the expression of ZNF746 was diminished by 62.5% (*p* < 0.001, Figure 4A). In addition, we also found that after 4 h of 6-OHDA exposure, the expressions of NRF1, TFAM, and PGC-1α began to decrease significantly, while the expression of ZNF746 began to increase (Figure 4B). However, CSS preprocessing significantly blocked this change (Figure 4B).

We also investigated the time-dependent changes in the expression of ZNF746, PGC-1α, NRF1, and TFAM proteins by CSS-only treatment. After 12 h of treatment, Western blot analysis showed that ZNF746 protein expression was reduced by 82.7% (*p* < 0.001, Figure 4C). However, the expression of PGC-1α protein was augmented by 3.9-fold (*p* < 0.001). NRF1 and TFAM upregulated by 2.4-fold (*p* < 0.001) and 3.4-fold (*p* < 0.001), respectively (Figure 4C). Moreover, as shown in Figure 4C, the expression of ZNF746 began to decrease after 2 h of CSS treatment and remained at a low level after 8 h. In contrast, the expression of PGC1-α started to increase after 4 h and remained high after 8 h. The expression levels of NRF1 and TEAM began to increase after 6 h and remained high after 8 h. The time points at which the expression levels of these proteins were altered by CSS treatment conformed to the order of the known ZNF746/PGC1-α/mitochondrial biogenesis axis. This shows that the expression of ZNF746, which was originally upregulated by 6-OHDA in Figure 4B, was effectively inhibited by CSS, maintaining the high expression of PGC1-α, NRF1, and TEAM. Therefore, mitochondrial biogenesis is promoted to avoid ROS production and apoptosis caused by 6-OHDA.

### 3.5. Parkin-Dependent Ubiquitination Degradation of ZNF746 Blocked by 6-OHDA Is Enhanced by CSS Pretreatment

**Figure 5:**
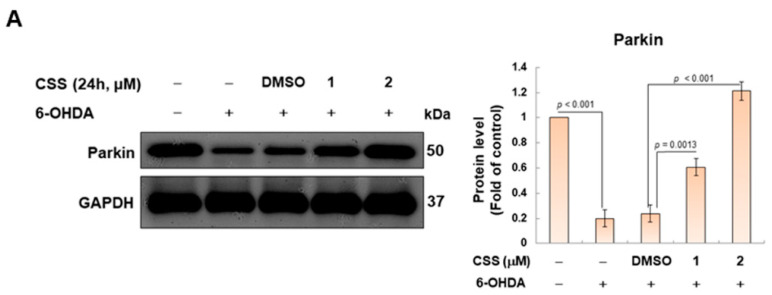

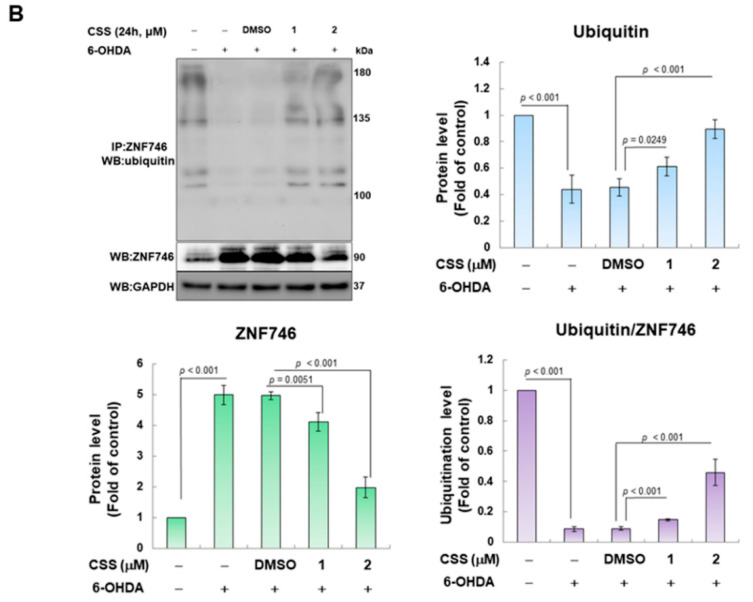
CSS enhances the parkin-dependent ubiquitination degradation of ZNF746 in 6-OHDA-exposed SH SY5Y cells. SH-SY5Y cells were pretreated with CSS for 24 h and then exposed to 6-OHDA (100 μM) for 12 h. (**A**) Protein expression of parkin was quantified by Western blotting. The protein loading control was GAPDH. (**B**) The degree of ubiquitination of ZNF746 was determined using immunoprecipitation with ZNF746 antibody and Western blot analysis with ubiquitin antibody. Signal intensity was quantified with ImageJ software. The addition of cell extracts was normalized using GAPDH.

We confirmed in this study that CSS treatment can reduce the expression of ZNF746 in SH-SY5Y cells and thus inhibit the toxicity of 6-OHDA. The parkin protein is one of the main regulators of ZNF746 expression. It can specifically promote the ubiquitination and degradation of ZNF746 [38]. Therefore, we wanted to confirm the effect of CSS treatment on parkin-dependent ubiquitination of ZNF746 by immunoprecipitating with ZNF746 antibody and then measuring the degree of ubiquitination of ZNF746. The study showed that 6-OHDA treatment reduced parkin expression by 80% in SH-SY5Y cells compared with the control group (*p* < 0.001, Figure 5A). However, under the pretreatment of 2 μM CSS, the expression of parkin increased by 5.0-fold compared with the DMSO/6-OHDA group (*p* < 0.001, Figure 5A). We further found that 6-OHDA exposure reduced the ubiquitination of ZNF746 by 91.2% (*p* < 0.001; the level of ubiquitination was normalized by the expression of ZNF746, Figure 5B). In contrast, CSS pretreatment significantly enhanced the ubiquitination of ZNF746 by 5.1-fold (Figure 5B). The above results indicated that CSS promoted the ubiquitination and degradation of ZNF746 by parkin.

### 3.6. Parkin RNAi Abrogates CSS Ability to Prevent 6-OHDA-Induced Downregulation of Mitochondrial Biogenesis Proteins and Activation of Apoptotic Proteins

**Figure 6:**
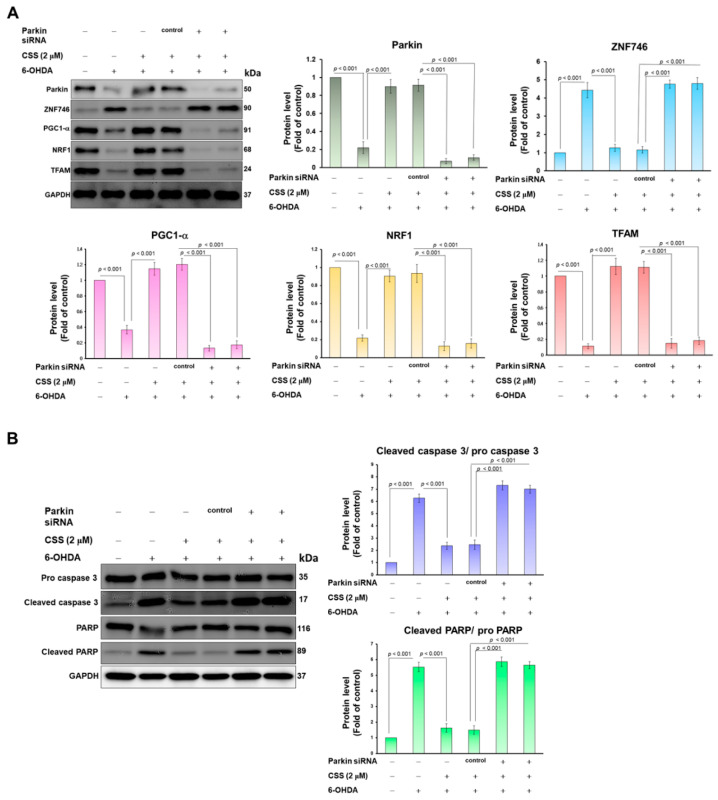
Downregulation of parkin abolishes the prevention effect of CSS on 6-OHDA-induced gene repression of mitochondrial biogenesis and promotion of apoptosis. (**A**) SH-SY5Y cells were delivered with control or parkin siRNA for 24 h. This was followed by CSS (2 μM) pretreatment for 24 h and 6-OHDA (100 μM) exposure for 12 h. Finally, Western blot was used to measure the protein expression of parkin, ZNF746, PGC-1α, NRF1, and TFAM. GAPDH acts as a protein loading control. (**B**) As described in (**A**), to measure the expression and activity of apoptosis-related proteins caspase 3 and PARP using Western blotting. GAPDH was used as a loading control.

Because our experiments confirmed that ZNF746 will be ubiquitinated and degraded under CSS pretreatment, we want to further confirm that this is due to the increased expression of parkin leading to the ubiquitination of ZNF746 and the up-regulation of downstream mitochondrial biogenesis-related genes. After 24 h of parkin RNAi transfection, CSS pretreatment was performed for 24 h followed by 12 h of 6-OHDA exposure. The results of Western blot revealed that compared with the RNAi control group, parkin RNAi treatment could reduce the expression of parkin by 92.3% (*p* < 0.001) (Figure 6A). We found that 6-OHDA-induced upregulation of ZNF746 and downregulation of PGC-1α reversed by CSS pretreatment were abolished by knockdown of parkin siRNA (Figure 6A). In addition, the properties of CSS which increased the expression of mitochondrial biogenesis-related genes (NRF1 and TFAM) (Figure 6A) and repressed expression of pro-apoptosis-related genes (Figure 6B) in 6-OHDA-exposed cells was inhibited by parkin siRNA knockdown. Therefore, this shows that the protective mechanism of CSS against 6-OHDA-induced mitochondrial biogenic damage and apoptosis in SH-SY5Y cells needs to prevent the expression of ZNF746 by activating parkin and then increase the activity of PGC-1α.

### 3.7. The Expression of Endogenous MiR-181a Induced by 6-OHDA Was Inhibited by CSS Pretreatment, Which Promoted the Increase in Parkin Level

**Figure 7:**
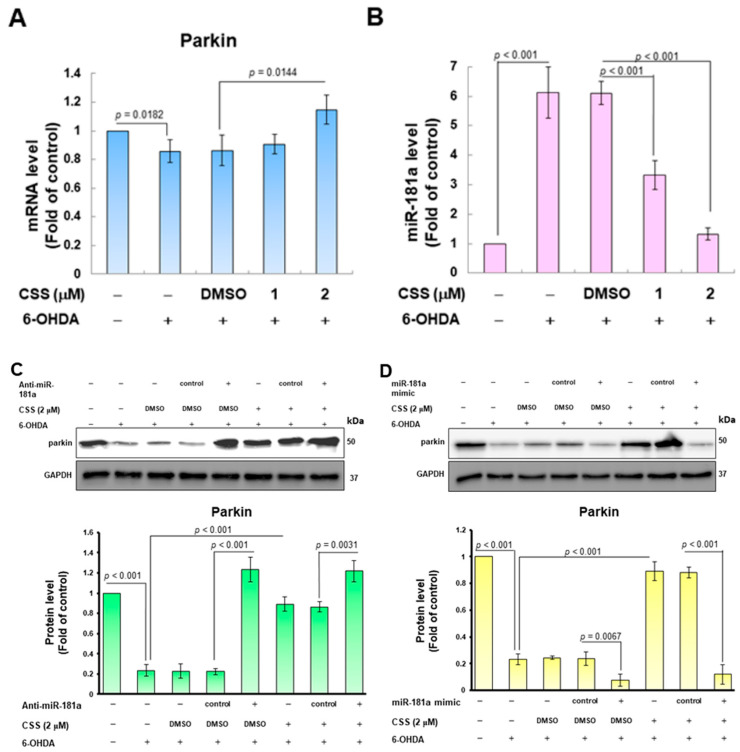
CSS prevents parkin downregulation by inhibiting 6-OHDA-induced miR-181a expression in SH-SY5Y cells. (**A**) The mRNA expression level of parkin in CSS-pretreated 6-OHDA-exposed SH-SY5Y cells was analyzed by RT-qPCR. (**B**) The level of miR-181a in (**A**) was analyzed by RT-qPCR. (**C**,**D**) SH-SY5Y cells were transfected with (**C**) anti-miR-181a (miR-181a inhibitor) or (**D**) miR-181a mimic (mature miR-181a) for 24 h. Next, cells were pretreated with CSS for 24 h and then exposed to 6-OHDA for 12 h. The expression of parkin was analyzed by Western blot. Internal loading control is GAPDH.

To more precisely confirm the possible mechanism by which CSS reversed 6-OHDA-induced inhibition of parkin activity, we first analyzed the expression of parkin mRNA after CSS pretreatment and 6-OHDA exposure. According to the results of the RT-qPCR assay, the level of parkin mRNA was slightly increased compared with the DMSO/6-OHDA group. Parkin mRNA was amplified 1.3-fold (*p* = 0.0144) under 2 μM CSS group (Figure 7A). Parkin expression could be inhibited by blocking the translation or promoting the degradation of mRNA by multiple endogenous miRNAs. The most prominent parkin-targeted specific miRNAs included miR181a [39]. Here, we hypothesize that CSS regulates the expression of miR181a. The results showed that when the cells were exposed to 6-OHDA, the expression of miR181a was significantly increased by 6.1-fold in RT-qPCR analysis (*p* < 0.001, Figure 7B). However, after 2 μM CSS pretreatment, the expression level of miR181a was significantly reduced by 78.2% compared with the DMSO/6-OHDA group (*p* < 0.001, Figure 7B). This is consistent with previous results of increased parkin protein expression (Figure 5A). To check the effect of miR-181a in the CSS-activated parkin pathway, we employed anti-miR181a (miR181a inhibitor) to inhibit endogenous miR181a. Compared the anti-miR181a group with the anti-miR control group, the Western blot results showed that the expression of parkin protein in 6-OHDA-exposed cells was significantly increased (*p* < 0.001, Figure 7C). We also found that 2 μM CSS pretreatment had no further effect on increasing parkin expression (Figure 7C). Furthermore, we overexpressed mature miR181a in SH-SY5Y cells using a miR181a mimic. The results revealed that miR181a mimic transfection abolished the ability of CSS to alleviate the downregulation of parkin caused by 6-OHDA exposure (Figure 7D). These statistics suggest that the character of miR-181a is a more upstream event on the CSS-mediated parkin/ZNF746/PGC1-α axis.

### 3.8. Degeneration of DA Neurons in 6-OHDA-Exposed Caenorhabditis Elegans Animal Models Can Be Reduced by CSS Pretreatment

**Figure 8:**
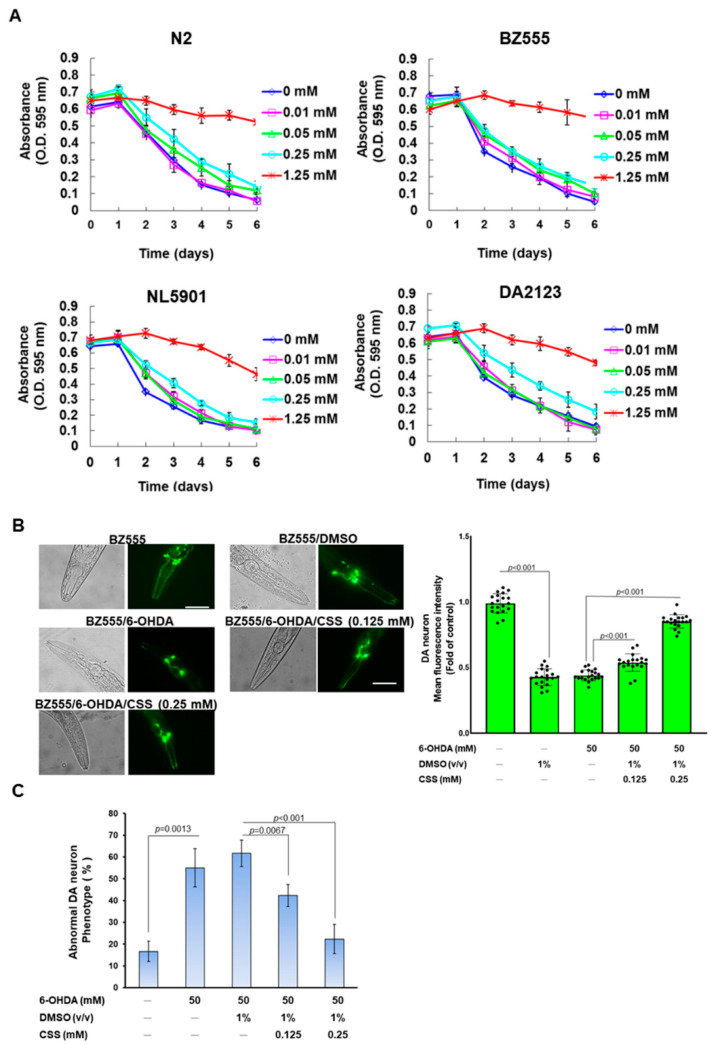

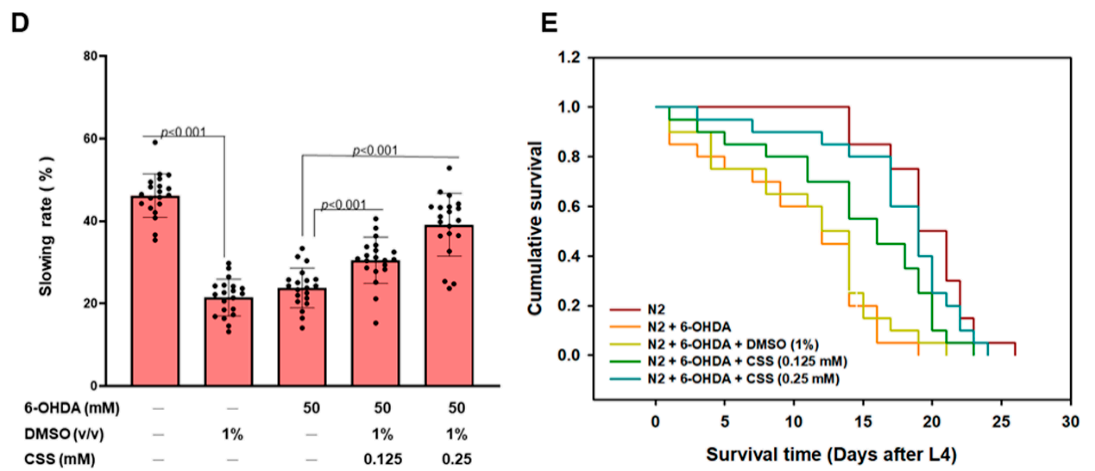
6-OHDA-caused degeneration of DA neurons, deficits in food-sensitive behavior, and shortened lifespan in *C. elegans* can be prevented by CSS pretreatment. (**A**) A food clearance test is used to determine the appropriate dose of CSS to treat nematodes. L1-stage nematodes (approximately twenty) of each strain were incubated on 96-well plates in an S medium containing *Escherichia coli* OP50 (OD_600_ = 0.6) and CSS for 6 days (20 °C). The OD value of each well was recorded daily. (**B**–**D**) L1 stage nematodes (BZ555 or N2) were grown to the L3 stage in culture plates with or without CSS. They were subsequently exposed to 6-OHDA solution (50 mM) for 1 h and then transferred to new plates with or without CSS for further incubation. The evaluation was performed 3 days later. (**B**) Representative fluorescent plots of GFP expression in the head of BZ555 nematodes indicated integrity in DA neurons. The solid black dots (•) represent the numerical distribution of individual samples. Scale bar = 50 µm. The fluorescence intensity of GFP images was quantified using ImageJ software. (**C**) Scoring for the phenotype of DA neuron deficits (disrupted axonal fluorescence signal or punctate degeneration) in (**B**) experiments. The percentage of nematodes with DA neuron deficient phenotype is shown in a bar graph. (**D**) Food sensitivity behavior assay was used to assess the function of DA neurons in *C. elegans* (N2). A total of 20 nematodes were evaluated per group. The solid black dots (•) represent the numerical distribution of individual samples. (**E**) L1 stage N2 nematodes were developed to the L3 stage in culture plates with or without CSS. They were subsequently exposed to 6-OHDA solution and then moved to new plates with or without CSS for further incubation. The number of surviving nematodes was calculated daily (20 nematodes per group).

To further evaluate the in vivo utility of CSS for PD-related neuroprotection, we used a simple *C. elegans* model organism (nematodes). First, we performed a food clearance test to obtain the optimal dose of CSS treatment. The results showed that when the medium was added up to 1.25 mM CSS, the food clearance curves of each strain of nematodes were significantly flattened compared with the untreated group (Figure 8A). These reflect that CSS at 1.25 mM is toxic to the physiology of nematodes. Since the food clearance curves of the individual strains were not meaningfully repressed below 0.25 mM CSS (Figure 8A), we treated nematodes with up to 0.25 mM CSS in subsequent tests.

Next, we used fluorescence microscopy to analyze the GFP-expressing DA neurons of BZ555 nematodes and then evaluate the protective ability of CSS against 6-OHDA exposure-induced damage to DA neurons. First, we confirmed that 6-OHDA exposure resulted in a marked decrease in the fluorescence intensity of DA neurons in the head of three pairs, representing impairment and degeneration of DA neurons (Figure 8B). The mean fluorescence intensity was reduced by 56.8% (*p* < 0.001) in the 6-OHDA group compared to the control group (Figure 8B). However, GFP fluorescence intensity in 6-OHDA-exposed nematodes could be enhanced in a dose-dependent manner by CSS pretreatment (Figure 8B). Pretreatment with 0.25 mM CSS effectively increased the fluorescence intensity by 1.9-fold (*p* < 0.001) compared to the DMSO/6-OHDA group (Figure 8B). Furthermore, the ratio of abnormal phenotypes of DA neurons was significantly increased by 3.3-fold in the 6-OHDA group compared with the control group (*p* = 0.0013) (Figure 8C). In the 0.25 mM CSS group compared with the DMSO/6-OHDA group, the phenotype of DA neuron degeneration was reduced by 63.9% (*p* < 0.001) (Figure 8C).

### 3.9. Dopamine-Mediated Deficits in Food-Sensitive Behaviors Induced by 6-OHDA Exposure in C. elegans Can Be Improved by CSS Pretreatment

Dopamine-mediated food-sensitivity behavior in *C. elegans* correlates positively with DA neuron function. Nematodes reduce the frequency of S-shaped body bending (movement speed) for high feeding efficiency when contacting food. We found that wild-type N2 worms had a 46.2% slowing rate (quantitative representation of changes in bending frequency) after exposure to bacterial lawns compared with empty lawns (Figure 8D). In addition, the slowing rate of N2 worms was significantly reduced by 53.5% in the 6-OHDA group compared to the control group (*p* < 0.001, Figure 8D). However, this slowing rate was restored by CSS pretreatment. Compared with the DMSO/6-OHDA group, the slowing rate increased 1.6-fold in the 0.25 mM CSS group (*p* < 0.001, Figure 8D). The above results showed that the functional deficit of DA neurons caused by 6-OHDA could be alleviated by CSS pretreatment.

### 3.10. The Lifespan of C. elegans Was Shortened by 6-OHDA Toxicity Can Be Extended by CSS Pretreatment

6-OHDA toxicity shortens the lifespan of nematodes [33]. Therefore, we wanted to assess whether the lifespan of nematodes shortened by 6-OHDA toxicity could be improved by pretreatment with CSS. The results showed that N2 nematodes lived shorter in the 6-OHDA group compared to the control group. Estimates from the lifespan cumulative survival model (Kaplan–Meier method) showed that the average survival time of 6-OHDA-exposed nematodes was 10.5 ± 2.2 days, which was significantly shortened (*p* < 0.001) compared with the control group (19.6 ± 1.6 days, Figure 8E). CSS pretreatment augmented the shortened lifespan of nematodes in a dose-dependent manner. The lifespan of nematodes in the 0.25 mM CCS group was extended to 17.6 ± 1.4 days (*p* < 0.001, Figure 8E). Therefore, the shortened lifespan caused by 6-OHDA could be improved by CSS pretreatment.

### 3.11. CSS Pretreatment Reduces the Level of Reactive Oxygen Species and Reverses the Downregulation of Pdr-1/Parkin, F45E4.9/TFAM, and SKN-1A/NRF1 in 6-OHDA-Exposed N2 Nematodes

**Figure 9:**
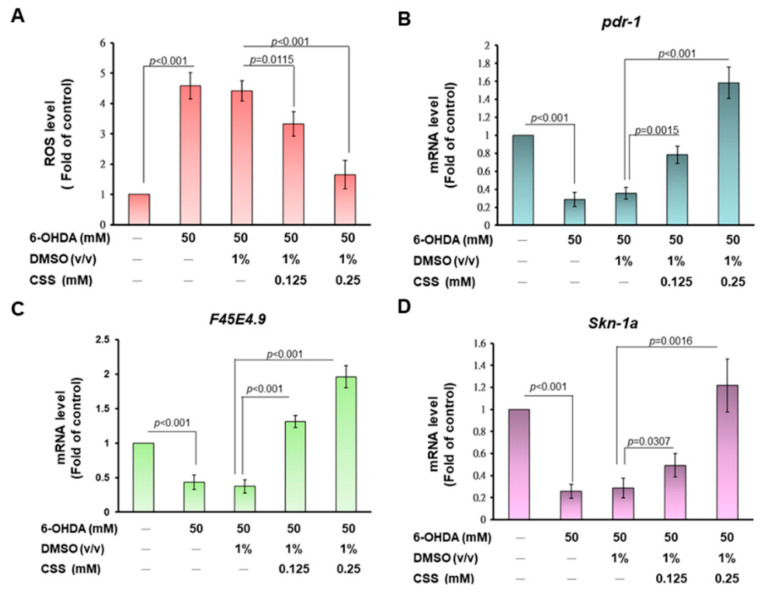

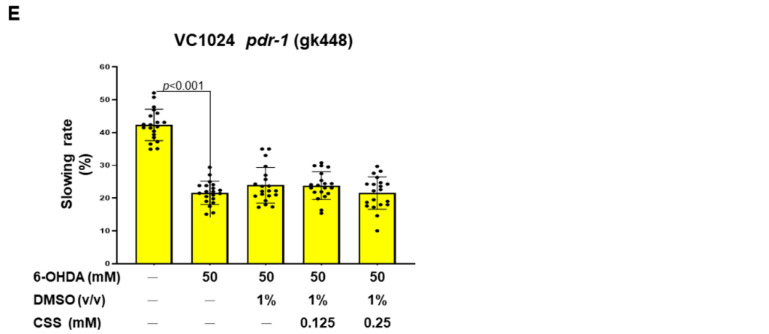
CSS pretreatment diminished the reactive oxygen species yield in 6-OHDA-exposed N2 nematodes by promoting the mRNA expression of pdr-1, F45E4, and Skin-1a. L1-stage N2 nematodes were grown in plates with or without CSS. At the L3 stage, nematodes were exposed to 6-OHDA for 1 h and then moved to fresh plates with or without CCS for 3 days. (**A**) ROS levels were measured using an H2DCFDA probe in 30 randomly selected nematodes in each group in the wells of a 96-well plate. (**B**–**D**) Quantification of the expression of pdr-1 (**B**), F45E4 (**C**), and Skin-1a (**D**) in each group of N2 nematodes by RT-qPCR. (**E**) Food sensitivity behavior assay was used to assess the function of DA neurons in VC1024 strain of *C. elegans*. A total of 20 nematodes were evaluated per group. The solid black dots (•) represent the numerical distribution of individual samples.

We further wanted to evaluate whether CSS would lessen ROS levels in 6-OHDA-exposed nematodes. ROS levels in nematodes increased 4.6-fold (*p* < 0.001) after exposure to 6-OHDA compared with the control group (Figure 9A). CSS pretreatment dose-dependently reduced the level of ROS in 6-OHDA-exposed nematodes. Pretreatment with 0.25 mM CSS decreased ROS levels by 62.7% (*p* = 0.0020) compared with the DMSO/6-OHDA group (Figure 9A).

According to the previous experimental results, we want to know whether the neuroprotective function of CSS in the nematode model of PD is the same as in the SH-SY5Y cell model by enhancing the activity of parkin (the orthologue in *C. elegans* is pdr-1) to promote expression of TFAM (ortholog in *C. elegans* is F45E4.9) and NRF1 (ortholog in *C. elegans* is SKN-1A). Finally, the mitochondrial biosynthetic pathway was activated to improve the 6-OHDA-induced apoptosis of DA neurons. The results of RT-qPCR showed that 6-OHDA exposure could inhibit the mRNA expression of pdr-1 (*p* < 0.001, Figure 9B), F45E4.9 (*p* < 0.001, Figure 9C), and skn-1a (*p* < 0.001, Figure 9D) in nematodes. Conversely, compared with the DMSO/6-OHDA group, CSS pretreatment dose-dependently increased the mRNA expressions of pdr-1, F45E4.9, and skn-1a in 6-OHDA-exposed nematodes, respectively (Figure 9B–D). Under 0.25 mM CSS pretreatment, the mRNA levels of pdr-1, F45E4.9, and skn-1a increased by 4.4-fold (*p* < 0.001, Figure 9B), 5.3-fold (*p* < 0.001, Figure 9C), and 4.2-fold (*p* < 0.001, Figure 9D). Finally, we used the mutant *C. elegans* of VC1024 (pdr-1 loss-of-function) to verify our observations. The results showed that CSS pretreatment did not improve the dopamine-related food-sensitive behavioral deficits induced by 6-OHDA exposure in VC1024 nematodes. This confirmed the key role of pdr-1 in the CSS-mediated anti-PD pathway.

### 3.12. CSS Reduces α-Synuclein Accumulation by Promoting Autophagy Activity

**Figure 10:**
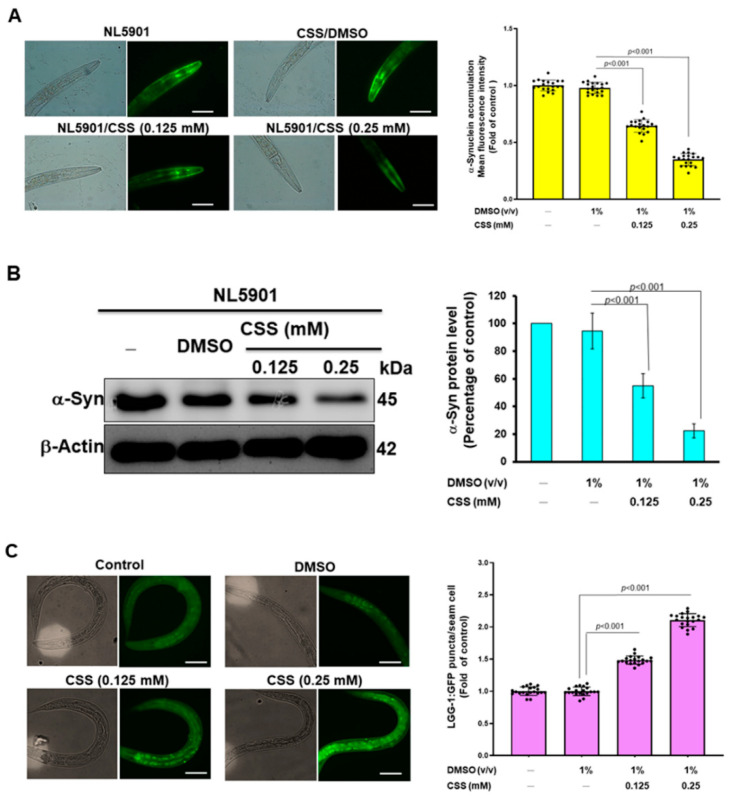
CSS treatment reduces α-synuclein accumulation in muscle cells of NL5901 nematodes. This may be achieved via upregulation of parkin-associated autophagy activity. (**A**) The accumulation of α-synuclein in muscle cells of NL5901 nematodes treated with CCS for three days (from L1-stage) was observed by fluorescence microscopy and quantified by ImageJ software. The solid black dots (•) represent the numerical distribution of individual samples. (**B**) The nematodes of (**A**) were subjected to Western blot analysis to determine the expression of α-synuclein and quantified using ImageJ software. β-actin acts as an internal loading control. (**C**) The L1 stage of DA2123 nematodes was treated with CSS for 3 days. Next, the number of green fluorescence dots formed by the LGG-1 promoter-derived GFP-fused LGG-1 protein in seam cells was analyzed by fluorescence microscopy. The solid black dots (•) represent the numerical distribution of individual samples.

In sporadic or hereditary PD, a common hallmark within DA neurons is the abnormal accumulation of α-synuclein. Here, we also assessed whether CSS had the ability to ameliorate abnormal accumulation of α-synuclein. As an α-synuclein accumulation model, we used NL5901 nematodes. Their muscle cells are overexpressed and accumulate YFP-fused human α-synuclein. They also develop symptoms of paralytic dyskinesia similar to PD. The quantification of fluorescence intensity in nematodes showed that the accumulation of α-synuclein in muscle cells could be dose-dependently reduced by CSS pretreatment (Figure 10A). Compared to the DMSO group, we found that α-synuclein accumulation was reduced by 64.4% in the 0.25 mM CSS group (*p* < 0.001, Figure 10A). Furthermore, Western blot revealed that the level of α-synuclein was dose-dependently diminished by CSS treatment. In the 0.25 mM CCS group, the level of α-synuclein was reduced by 76.5% compared with the DMSO group (*p* < 0.001, Figure 10B). Earlier studies have demonstrated that the activities of parkin can upregulate the activity of autophagy to block abnormal protein accumulation in cells [40]. Therefore, we further wanted to use the transgenic nematode strain DA2123 to assess the effect of CSS on autophagy activity in nematodes. The hypodermal seam cells of this nematode express the LGG-1 promoter-derived human LC3 orthology LGG-1 protein fused to GFP. Therefore, the formation of green fluorescent dots in cells is positively correlated with the formation of autolysosomes (It represents autophagy activity). The results of the fluorescence microscopy analysis showed that the activity of nematode autophagy was dose-dependently increased by CSS treatment (Figure 10C). Under 0.25 mM CSS treatment, the average number of GFP dots per nematode increased 2.1-fold compared with the DMSO group (*p* < 0.001, Figure 10C). The above results indicated that the autophagy activity in nematodes could be increased by CSS treatment.

## 4. Discussion

At present, the treatment of PD can only alleviate the symptoms of the disease. The development of related drugs is mainly based on oral or injectable dopamine substitutes or their degradation inhibitors [41]. Finding strategies that can prevent, delay, or reverse the degeneration of dopamine neurons, including the efficacy of phytochemicals in the treatment of PD, has been a hot research focus. Our study found that chiisanoside (CSS) from the leaves of *Acanthopanax sessiliflorus* can reduce ROS production and inhibit apoptosis in the neurotoxin 6-OHDA-treated SH-SY5Y cell model (in vitro model), thereby alleviating DA neuron degeneration, DA-mediated food-sensitive behavioral deficits, and shortened lifespan in an animal model of *C. elegans* (in vivo model). This is the first study on the efficacy of CSS in PD treatment.

ATP production, Ca^2+^ homeostasis, and apoptosis, which are closely related to mitochondria, play a major role in the survival and activity of neurons [42]. These explain the importance of fine-tuning the mitochondrial pool of newly synthesized mitochondrial components for the replenishment of mitochondrial pools and the degradation of old or damaged mitochondria to continuously adapt the mitochondrial network to various needs [43]. Therefore, in the process of neurodegenerative diseases and aging, one of the important factors is mitochondrial dysfunction. PD is associated with the dysregulation of the mitochondrial quality control system within DA neurons in the substantia nigra. Homeostasis of healthy mitochondria is achieved by fine regulation of molecules involved in mitochondrial biogenesis, mitophagy, and mitochondrial dynamics [44]. This study shows that 6-OHDA-induced damage and apoptosis of SH-SY5Y cells can be improved by enhancing mitochondrial biogenesis via CSS treatment.

One of the major regulators of mitochondrial biogenesis is PGC-1α, and the PGC-1α-NRF1 (NRF2)-TFAM pathway manipulates mitochondrial biogenesis at the transcriptional level [5]. Studies have shown that the development of PD is related to the lack of the expression of PGC-1α in the substantia nigra of patients [7]. In vitro studies have shown that the knockdown of PGC-1α reduces mitochondrial membrane potential, mitochondrial mass, and the expression of its downstream target genes, including TFAM and NRF. Increasing PGC-1α levels upregulated the expression of nuclear-encoded subunits of the mitochondrial respiratory chain and ameliorated DA neuronal apoptosis induced using rotenone and α-synuclein mutant [45]. PGC-1α is also related to mitochondrial fission/fusion and mitophagy. Increasing expression of PGC-1α can inhibit mitochondrial fission [46], promote mitophagy [47], and strengthen the activation of antioxidant systems such as glutathione [48]. In vivo experiments showed that the tetramethylpyrazine (TMP) derivative enhanced PGC-1α pathway can attenuate the loss of tyrosine hydroxylase-positive neurons in substantia nigra pars compacta of 6-OHDA lesion mice and dopaminergic nerve fibers in the striatum, and also increased the concentration of internal DA and its metabolites, finally improving the deficits in motor coordination and rotational behavior [49]. In addition, it is known that a variety of small molecules from natural sources such as vitamin K2 [50], urolithin A [51], carnosic acid [52], pyrroloquinoline quinone [53], allicin [54], etc., also have the potential to regulate PGC-1α. Interestingly, exercise in a unilateral PD rat model prevented the decrease in PGC-1α and NRF-1 expression [55]. In the 1-Methyl-4-phenyl-1,2,3,6-tetrahydropyridine (MPTP)-induced PD mouse model, hyperbaric oxygen therapy can increase PGC-1α expression and improve DA neuron death and motor deficits [56]. In this study, we found that CSS ameliorated the expression of PGC-1α, NRF1, and TFAM that was decreased using 6-OHDA.

Studies have shown that one of the transcriptional repressors of PGC-1α is ZNF746. Chromatin immunoprecipitation results have also demonstrated that ZNF746 binds to the PGC-1α promoter region in SH-SY5Y cells and the mouse/human brain [13]. ZNF746 primarily binds to the insulin response sequence (IRS) on the promoter of PGC-1α, thus causing the repression of PGC-1α and its target genes. Accumulating evidence now points to a critical role of ZNF746 in the observed defects in mitochondrial biogenesis in PD pathogenesis [57,58]. Abnormal expression of ZNF746 is considered to be one of the risk factors for PD. ZNF746 protein was significantly increased in the substantia nigra and striatum of some PD patients [13]. Furthermore, overexpression of ZNF746 in the mouse brain resulted in decreased mitochondrial respiration and number, a defect in mitochondrial biogenesis due to downregulation of PGC-1α and NRF1 expression [13]. ZNF746 overexpression also induces cellular aging [59]. Furthermore, overexpression of ZNF746 inhibits transketolase and then down-regulates the pentose phosphate pathway, increases intracellular H_2_O_2_ production, reduces NADPH and GSH levels, and finally triggers the main promoter of glycolysis HIF-1α, leading to the death of DA neurons [60].

Recent studies have shown that farnesylation of ZNF746 in the substantia nigra of PD patients is reduced, indicating that the reduction in farnesylation of ZNF746 may be related to PD. Farnesol is an inhibitor of ZNF746. Farnesol can increase the farnesylation of ZNF746, thereby reducing the occupancy of ZNF746 on the PGC-1α promoter to prevent the expression inhibition of PGC-1α. Farnesol prevents DA neuron loss and behavioral deficits in both adult conditional parkin knockout mice and the α-synuclein fibril model of sporadic PD [38]. Moreover, ZNF746-mediated transcriptional repression is also ameliorated by SUMO-targeted ubiquitin ligase RNF4-mediated proteasomal degradation of SUMO-targeted ubiquitin [61]. The phosphorylation of ZNF746 by PINK1 promotes the ubiquitination and clearance of ZNF746 by parkin, leading to the activation of the PGC-1α promoter [10]. In this study, we found that 6-OHDA could enhance the protein expression of ZNF746 in SH-SY5Y cells. However, CSS pretreatment effectively reduced the effect of 6-OHDA on ZNF746 protein induction. The downregulation effect of CSS on the protein level of ZNF746 may thus promote the upregulation of PGC-1α, NRF1, and TFAM protein expression. In this study, we found that CSS did not affect farnesylation, SUMOylation, and phosphorylation of ZNF746, leading to its degradation, but was affected by ubiquitination.

Studies have shown that the degradation of ZNF746 is tightly regulated by parkin [62]. Parkin is an E3 ubiquitin ligase that plays a critical role in the ubiquitination involved in the ubiquitin-proteasome system and regulation of protein activity/localization [63]. The loss of parkin function will affect mitochondrial fission/fusion, transport, and mitophagy and finally lead to the destruction of mitochondrial quality control [64]. The report showed that loss-of-function mutations in parkin cause autosomal recessive PD. There is also evidence that parkin is inactivated in sporadic PD [65]. The loss of DA neurons accompanied by increased ZNF746 protein and decreased PGC-1α and mitochondrial biogenesis were found in the brains of PD patients with parkin mutations [62]. Studies have shown that the knockdown of parkin in adult mouse brains leads to reductions in mitochondrial size, number, and protein markers, which are prevented by the knockdown of ZNF746 [66]. Previous studies have shown that parkin is phosphorylated and inactivated by c-Abelson kinase in a model of α-synuclein-induced neurodegeneration. This leads to the accumulation of ZNF 746 and is strongly associated with α-synuclein-induced neurodegeneration [62]. Loss of parkin activity in mouse and human DA neurons leads to accumulation of ZNF746 and inhibition of processes requiring ubiquitination and targeting of NOD-like receptor protein (NLRP3) for proteasomal degradation. Finally, it was initiated by mitochondria-derived reactive oxygen species generation, leading to the assembly of the NLRP3 inflammasome [67]. In this study, immunoprecipitation analysis revealed that 6-OHDA exposure significantly reduced the ubiquitination of ZNF746. However, CSS pretreating reverses this situation. This result suggests that CSS can promote the degradation of ubiquitinated ZNF746 and thereby inhibit the accumulation of ZNF746 in cells. Furthermore, the knockdown of parkin using a specific siRNA abolished the inhibitory effect of CSS on 6-OHDA-induced elevation of ZNF746 and reduction in PGC-1α. Thus, we demonstrated that CSS promotes the increase in PGC-1α protein by promoting parkin-associated ubiquitination of ZNF746. In addition, the inhibition of parkin blocked CSS’s ability to improve 6-OHDA-caused downregulation of NRF1 and TFAM expression. Likewise, inhibition of caspase 3 and PARP proteolysis. Therefore, CSS-induced mitochondrial biogenesis and antiapoptotic effects are mainly related to the upregulation of parkin expression.

**Figure 11:**
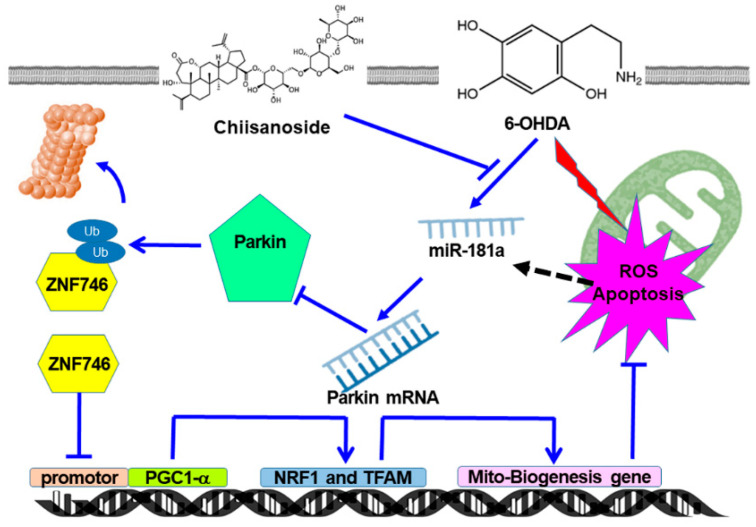
A diagram of the mechanism of action of CSS in this study. The function of CSS is mainly to promote TFAM- and NRF-related mitochondrial biogenesis by regulating the miR-181a/parkin/ZNF746/PGC-1α axis. Therefore, the oxidative stress and apoptosis of DA neurons caused by 6-OHDA are prevented.

Finally, we also demonstrated that 6-OHDA exposure upregulated miR-181a in SH-SY5Y cells, which targets parkin and suppresses parkin levels (Figure 11). CSS pretreatment can block the expression of miR-181a and enhance the activity of parkin. miR-181a is an endogenous regulator of mitochondrial dynamics-related genes such as parkin, p62/SQSTM1, and DJ-1 [39]. Downregulation of miR-181a in mitochondrial diseases such as microphthalmia with linear skin lesions and Leber’s hereditary optic neuropathy can enhance mitochondrial turnover in the retina by coordinating the initiation of mitochondrial biogenesis and mitophagy, protecting retinal neurons from cell death [68]. Thus, miR-181a is a novel inhibitor of mitophagy, and its knockdown accelerates the autophagic degradation of damaged mitochondria [69]. Studies have shown that mRNAs of genes for synaptic transmission, neurite outgrowth, and mitochondrial respiration targeted by miR-181 are broadly downregulated in aging and PD brains [70]. For example, miR-181a inhibits the expression of TFAM [71]. The overexpression of miR-181a exacerbates α-synuclein-induced loss of DA neurons in a mouse PD model, whereas miR-181a inhibition has neuroprotective effects [70]. In addition, PD risk-associated insecticides such as chlorpyrifos upregulate miR-181a expression to initiate pyroptosis and oxidative stress and down-regulate SIRT1/PGC-1α/Nrf2 signaling in the SH-SY5Y cell model [72]. The expression of miR-181a also inhibits neuronal or tumor cell proliferation and promotes apoptosis [73,74]. Therefore, the down-regulation of miR-181a expression by CSS can not only enhance the expression of parkin to promote mitochondrial biogenesis but also increase the ability of mitophagy, anti-oxidation, and anti-apoptosis.

DA neurons are more sensitive to pro-inflammatory mediators than other cell types [75]. In a previous report, CSS inhibited lipopolysaccharide-induced nitric oxide production and cyclooxygenase-2 expression by inhibiting the activation of NF-κB in RAW 264.7 macrophages [22]. CSS can also provide anti-inflammatory effects in Freund’s complete adjuvant-induced rat model and in the mouse model of acute liver injury induced using acetaminophen [76,77]. Therefore, CSS may reduce DA neuron damage in the brains of patients with PD by improving chronic inflammation. Studies have shown that a risk of PD is linked with metabolic-related diseases such as diabetes [78]. Yoshizumi et al. reported that CSS has the potential to act as a lipase inhibitor to improve obesity and thereby reduce body fat absorption [24]. Hence, CSS may also diminish the risk of PD by reducing the incidence of diabetes.

## 5. Conclusions

Taken together, CSS has neuroprotective properties in both in vitro and in vivo models of PD. The function of CSS is mainly to promote TFAM- and NRF-related mitochondrial biogenesis by regulating the miR-181a/parkin/ZNF746/PGC-1α axis. Thus, it prevents the degeneration of DA neurons caused by 6-OHDA-induced apoptosis and the accumulation of α-synuclein. CSS may have the potential to be developed as a prevention agent for PD.

## Data Availability

All data used and analyzed during the current study are available from the corresponding author upon reasonable request.
